# Descriptive distribution and phylogenetic analysis of feline infectious peritonitis virus isolates of Malaysia

**DOI:** 10.1186/1751-0147-52-1

**Published:** 2010-01-06

**Authors:** Saeed Sharif, Siti S Arshad, Mohd Hair-Bejo, Abdul R Omar, Nazariah A Zeenathul, Lau S Fong, Nor-Alimah Rahman, Habibah Arshad, Shahirudin Shamsudin, Mohd-Kamarudin A Isa

**Affiliations:** 1 Department of Veterinary Pathology and Microbiology, Faculty of Veterinary Medicine, Universiti Putra Malaysia, 43400 UPM Serdang, Selangor, Malaysia; 2 Department of Clinical Studies, Faculty of Veterinary Medicine, Universiti Putra Malaysia, 43400 UPM Serdang, Selangor, Malaysia; 3 University Veterinary Hospital, Faculty of Veterinary Medicine, Universiti Putra Malaysia, 43400 UPM Serdang, Selangor, Malaysia

## Abstract

The descriptive distribution and phylogeny of feline coronaviruses (FCoVs) were studied in cats suspected of having feline infectious peritonitis (FIP) in Malaysia. Ascitic fluids and/or biopsy samples were subjected to a reverse transcription polymerase chain reaction (RT-PCR) targeted for a conserved region of 3'untranslated region (3'UTR) of the FCoV genome. Eighty nine percent of the sampled animals were positive for the presence of FCoV. Among the FCoV positive cats, 80% of cats were males and 64% were below 2 years of age. The FCoV positive cases included 56% domestic short hair (DSH), 40% Persian, and 4% Siamese cats. The nucleotide sequences of 10 selected amplified products from FIP cases were determined. The sequence comparison revealed that the field isolates had 96% homology with a few point mutations. The extent of homology decreased to 93% when compared with reference strains. The overall branching pattern of phylogenetic tree showed two distinct clusters, where all Malaysian isolates fall into one main genetic cluster. These findings provided the first genetic information of FCoV in Malaysia.

## Findings

Feline infectious peritonitis (FIP) is a highly fatal disease of cats caused by generalized infection with a feline coronavirus (FCoV). FCoVs belong to subgroup 1a of Coronaviruses in the family *Coronaviridae*, order *Nidovirales*. Other members of this subgroup include porcine transmissible gastroenteritis virus, canine coronavirus, raccoon/dog coronavirus and Chinese ferret badger coronavirus [1,2]. FCoVs are enveloped, positive-strand RNA viruses with a large, capped and polyadenylated RNA genome of about 29 kb. The cap structure at the 5' end of genome is followed by an untranslated region (UTR). At the 3' end of the genome is another UTR of 275 nucleotides, followed by the poly (A) tail. The sequences of the both 3'- and 5'-UTR are important for RNA replication and transcription [3].

Two biotypes of FCoV are described in cats: feline infectious peritonitis virus (FIPV) and feline enteric coronavirus (FECV). Infection with FECV is usually unapparent or manifested by a transient gastroenteritis. In contrast, FIPV infection causes a fatal immune-mediated disease with a wide spectrum of clinical signs. FIP refers to the more common effusive (wet) form of the disease characterized by peritonitis and/or pleuritis. The effusive form is caused by complement-mediated vasculitis and results in inflammatory exudation into body cavities. In some FIP cases, partial cell-mediated immunity cause non-effusive (dry) form which is characterized by granulomatous involvement of various organs particularly central nervous system and eyes. However, the FIP forms can transform to each other [4-6]. It has been suggested that virulent FIPV arises by mutation from parental FECV in the individual, persistently infected host [4,7,8]. It is not yet clear which alterations in the FCoV genome are responsible for the generation of FIPV from FECV [3,6].

FIP occurs worldwide and is ubiquitous in virtually all cat populations [6]. The disease was reported as a major factor of kitten mortality in UK [9] and it is currently one of the leading infectious diseases causing death among young cats from shelters and catteries [6].

The first case of FIP in Malaysia was reported in 1981 [10] and the feature of cats with FIP were described in a retrospective study [11]. Antibodies against FCoVs were found in 100% of cats living in Malaysian catteries [12] and the virus was detected in 84% of healthy cats using RT-PCR [13]. In present study, a conserved region of 3'untranslated region (3'UTR) is used to detect FCoV and determine the descriptive distribution and phylogeny of local isolates in FIP-suspected cats.

Abdominal fluids and/or tissue samples of 28 cats suspected of having the effusive form of FIP were obtained from the University Veterinary Hospital, Universiti Putra Malaysia (UVH-UPM) over the period of three years (2007-2009). Ascitic fluids were diluted 1:10 in phosphate buffer solution (PBS), aliquoted and stored at -70°C until used. Organ samples were homogenized in 1:10 of PBS. Insoluble components were removed by centrifugation for 10 min at 3000 g and the supernatant fraction was collected and kept at -70°C. Two FCoV reference strains (FECV 79-1683; ATCC^® ^No.VR-989™ and FIPV79-1146; ATCC^® ^No. VR-216™) were used for RT-PCR optimization. Virus stocks were propagated in confluent Crandell Feline Kidney cells. The viruses were harvested when the infected cells showed 80% cytopathic effects. The virus suspension was freezed-thawed three times and stored at -70°C until used.

RNA was extracted from the infected cell culture supernatants and clinical samples using TRIZOL^® ^Reagent (Invitrogen, Carlsbad, California, USA) according to the manufacturer's instructions. The partial 3'UTR was amplified by RT-PCR using previously described primers [7]. One-step RT-PCR was performed using Access RT-PCR System and RNasin^® ^Ribonuclease Inhibitor (Promega, Madison, Wisconsin, USA). The reaction was optimized on a thermal cycler (MJ Research, Waltham, Massachusetts, USA). PCR products of 223 bp were analyzed using electrophoresis on a 2% agarose gel, stained with ethidium bromide and observed under UV light. PCR products of 10 positive cases were selected randomly, purified using PCR SV protocol (GENEALL^®^, Seoul, South Korea) and sequenced in both direction with the primers (Medigene, Selangor, Malaysia).

Data analysis was performed using Statistical Tables Calculator, which is available online at http://faculty.vassar.edu/lowry/odds2x2.html. Age, breed and gender differences were compared by calculating positivity rate, odds and 95% confidence intervals.

The RT-PCR assay amplified the target band in 25 out of 28 cats' samples (89%). Although, the PCR results must be interpreted in conjunction with clinical or pathological findings, detection of the virus in FIP-suspected cats may be useful to confirm FIP. Since FCoVs are ubiquitous in cats with high seroprevalence [5,6,12], PCR provides the obvious advantage over serology by directly detecting FCoV genome rather than documenting a previous immune system encounter with the coronavirus. The primers of this PCR assay were chosen from a highly conserved region of 3'UTR of the FCoV genome to detect most, if not all of the FCoV strains. The usefulness of these primers for a general screening test has been reported previously [14-16].

FCoV-positivity rate in cats younger than two years old (64%) was higher than older cats, but they are not significant. However, the result is consistent with other studies demonstrating higher incidence of FIP in cats below 2 years of age [5,11,14] and agree with the fact that FIP is a disease of young cats. Typical clinical cases are first appear during the postweaning period, but most deaths from FIP occur in cats 3-16 months of age [6].

Most of the FCoV-positive cats in our study were males (80%). Higher incidence of FIP among males was previously reported [14,17,18]. As the pathogenesis of the disease is still not fully understood, the relation of gender and incidence of FIP is not clear.

**Figure 1:**
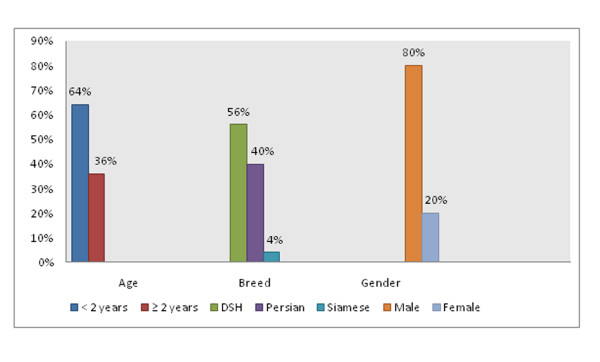
**Distribution of feline coronavirus positive cats categorized by age, breed and gender**. DSH: Domestic Short Hair

About 56% of FCoV-positive cases were DSH, 40% Persian, and 4% Siamese cats. In the present study, the majority of cats (96%) diagnosed with FIP were DSH or Persian. This finding is in accordance with a previous report on FIP in Malaysia showing that 69.7% and 27.3% of cats diagnosed with FIP were DSH and Persian cats, respectively [11]. However, these studies did not conclude that these two breeds were more susceptible to FIP because of limited variation in cat breeds presented at UVH-UPM and lack of clinical cases of FIP in different breeds in Malaysia. Furthermore, in a study on the prevalence of FIP in specific cat breeds, DSH and Persian cats were at low risk compared to others [18]. Age, breed and gender distribution in FCoV-positive cats are shown in Figure 1 and statistical analysis is summarized in Table 1.

**Table 1:** Statistical analysis of feline infectious peritonitis suspected cats tested for feline coronavirus (FCoV) by RT-PCR assay.

	**Criteria**	**No. of tested cats**	**No. of FCoV-positive cats**	**FCoV-positivity (%)**	**Odds**	**Odds Ratio**	**Confidence Interval**
	
**Age**	< 2 years	17	16	94	16	3.5556	0.2816 to 44.886
	≥ 2 years	11	9	82	4.5		
	
**Breed**	DSH	17	14	82	4.67	*	*
	Persian	10	10	100	*	*	*
	Siamese	1	1	100	*	*	*
	
**Gender**	Male	21	20	95	20	8	0.5985 to 106.9411
	Female	7	5	71	2.5		

DSH: Domestic Short Hair

* Insufficient number of cats to allow statistical calculations.

Out of 25 PCR positive cases, 10 isolates were selected for further sequence analyses. All 10 field isolates designated as UPM1C/07 to UPM10C/09 with accession no. FJ897745 to FJ897754, respectively were deposited in the GeneBank (Table 2). These sequences were aligned with published sequences of FCoV using ClustalW Multiple alignment (Bioedit version 7.0.9). The sequences of four Malaysian FCoV isolates which have been isolated from healthy cats in a previous study [19] were also included in the alignment (Table 2). Homology matrix and phylogenetic trees were constructed using Neighbor-Joining method (Bioedit) and TreeTop-Phylogenetic Tree Prediction (GeneBee-Molecular Biology Server available at http://www.genebee.msu.su). The phylogenetic trees were displayed in PHYLIP format including bootstrap values.

**Table 2:** List of feline coronavirus isolates and strains included in the sequence and phylogenetic analysis.

**No**.	Isolate/Strain	**Accession No**.	Origin	Reference
1	UPM1C/07	FJ897745	Malaysia	This paper
2	UPM2C/07	FJ897746	Malaysia	This paper
3	UPM3C/07	FJ897747	Malaysia	This paper
4	UPM4C/08	FJ897748	Malaysia	This paper
5	UPM5C/08	FJ897749	Malaysia	This paper
6	UPM6C/08	FJ897750	Malaysia	This paper
7	UPM7C/09	FJ897751	Malaysia	This paper
8	UPM8C/09	FJ897752	Malaysia	This paper
9	UPM9C/09	FJ897753	Malaysia	This paper
10	UPM10C/09	FJ897754	Malaysia	This paper
11	UPM28C/08	GQ233036	Malaysia	[19]
12	UPM29C/08	GQ233037	Malaysia	[19]
13	UPM30C/09	GQ233038	Malaysia	[19]
14	UPM31C/09	GQ233039	Malaysia	[19]
15	UU10	FJ938059	Netherlands	Unpublished
16	UU15	FJ938057	Netherlands	Unpublished
17	UU11	FJ938052	Netherlands	Unpublished
18	UU9	FJ938062	Netherlands	Unpublished
19	UU3	FJ938061	USA	Unpublished
20	UU2	FJ938060	USA	Unpublished
21	RM	FJ938051	USA	Unpublished
22	UCD11b-2b	FJ917535	USA	Unpublished
23	UCD11b-2a	FJ917534	USA	Unpublished
24	UCD11b-1b	FJ917533	USA	Unpublished
25	UCD11b-1a	FJ917532	USA	Unpublished
26	UCD11a-1b	FJ917531	USA	Unpublished
27	UCD11a-1a	FJ917530	USA	Unpublished
28	UCD17	FJ917527	USA	Unpublished
29	UCD14	FJ917524	USA	Unpublished
30	UCD13	FJ917523	USA	Unpublished
31	UCD5	FJ917522	USA	Unpublished
32	UCD12	FJ917521	USA	Unpublished
33	UCD11b	FJ917520	USA	Unpublished
34	UCD11a	FJ917519	USA	Unpublished
35	Black	EU186072	USA	[3]
36	NTU2/R/2003	DQ160294	Taiwan	Unpublished
37	UU16	FJ938058	Netherlands	Unpublished
38	UU5	FJ938056	Netherlands	Unpublished
39	UU8	FJ938055	Netherlands	Unpublished
40	UU7	FJ938053	Netherlands	Unpublished
41	UCD18b	FJ917529	USA	Unpublished
42	UCD18a	FJ917528	USA	Unpublished
43	UCD16	FJ917526	USA	Unpublished
44	UCD15a	FJ917525	USA	Unpublished
45	DF-2	DQ286389	USA	Unpublished
46	C1Je	DQ848678	UK	Unpublished
47	NTU156/P/2007	GQ152141	Taiwan	Unpublished
48	UU4	FJ938054	Netherlands	Unpublished
49	79-1146	DQ010921	USA	[21]
50	Wellcome	X90571	Netherlands	[22]
51	UCD1	X90575	USA	[22]
52	UCD	X90574	USA	[22]
53	TN406	X90570	Netherlands	[22]
54	UCD3a	FJ943761	USA	Unpublished
55	UCD2	X90576	USA	[22]
56	Dahlberg	X90572	Netherlands	[22]
57	UCD3	X90577	USA	[22]
58	UCD4	X90578	USA	[22]
59	NOR15	X90573	Netherlands	[22]
60	UCD12-1	FJ943766	USA	Unpublished
61	UCD6-1	FJ943772	USA	Unpublished
62	79-1683	X66718	USA	[23]

The sequences of ten local isolates showed 96% homology and when compared to published sequences of FCoV, the homology decreased to 93%. The homology between partial sequences of FCoV isolates from Malaysia were higher than those from different geographical origin (32 strains from USA, 13 strains from Netherlands, two strains from Taiwan, and one strain from UK). These findings support previous observations showing a correlation between different FCoV biotypes with similar geographic background [8].

**Figure 2:**
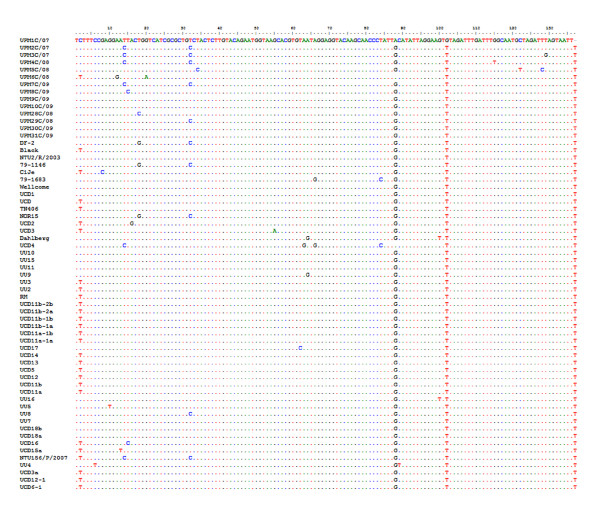
**Comparison of partial sequence of 3'UTR of Malaysian isolates and reference strains of feline coronaviruses**. Multiple alignments were performed using ClustalW Multiple alignment (Bioedit version 7.0.9). The sequences of the primers were removed from the alignment. Dots indicate identity.

Multiple sequence alignment showed a few point mutations and single-nucleotide deletions in the sequences of local isolates (Figure 2). These findings indicate single nucleotide polymorphisms (SNPs) in FCoVs as described previously [6,20]. No particular pattern of mutation or deletion was found in this part of FCoVs genome.

**Figure 3:**
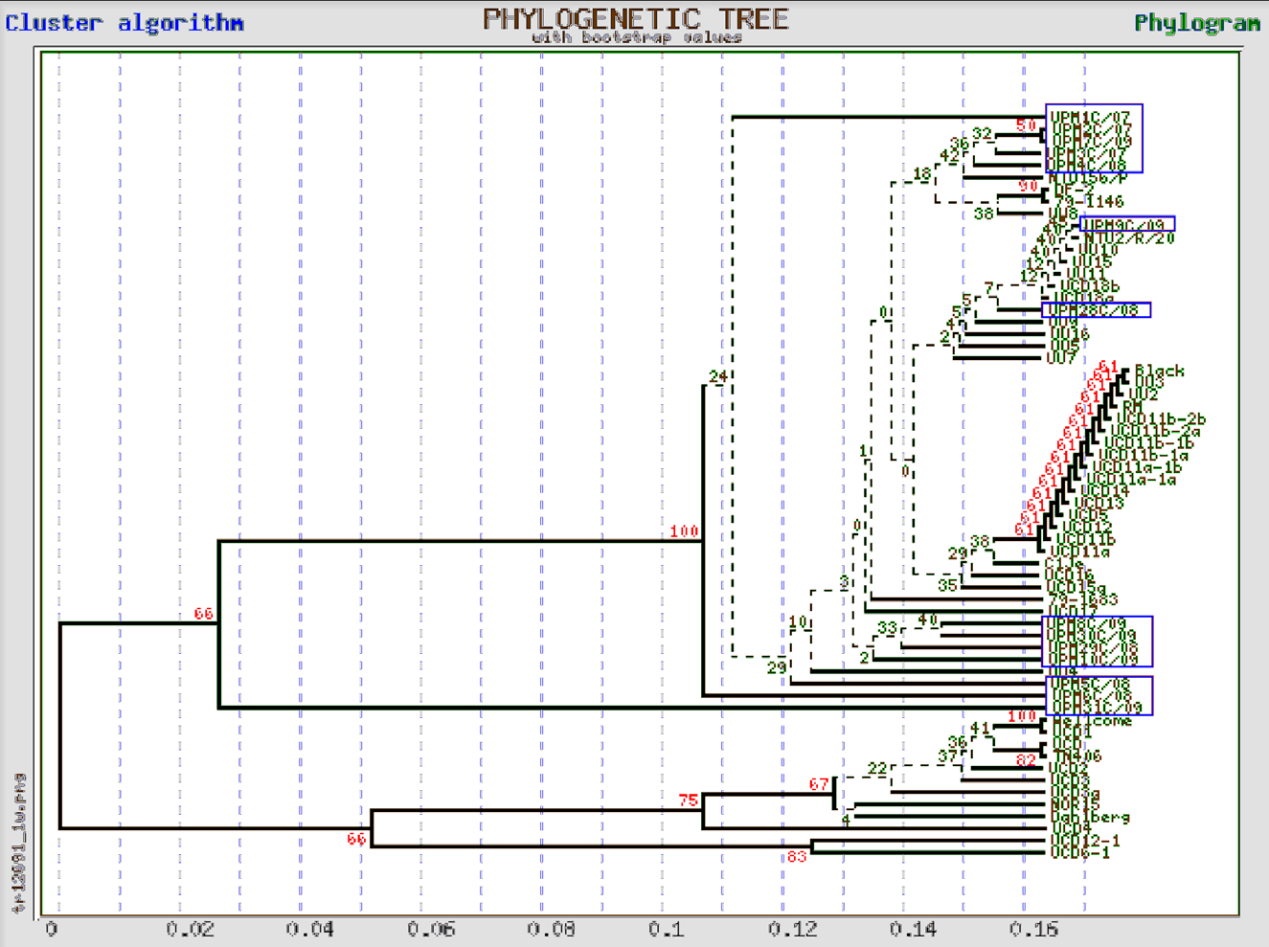
**Phylogenetic tree based on partial sequence of feline coronaviruses**. Malaysian isolates are marked by frames and categorized in one main cluster. The tree constructed by Tree Top-Phylogenetic Tree Prediction (GeneBee - Molecular Biology Server). The tree is displayed in PHYLIP format with bootstrap values.

**Figure 4:**
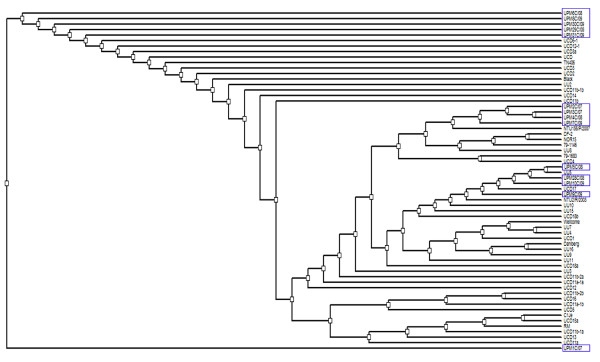
**Neighbor phylogenetic tree of feline coronavirus (FCoV) strains and isolates**. Partial sequences of FCoVs were subjected to DNADist version 3.5c and the result showed as a neighbor-joining algorithm (Bioedit version 7.0.9). Malaysian isolates are marked by frames.

Phylogenetic tree constructed by cluster algorithm showed that the sequences were genetically separated in two distinct clusters; all local sequences fell into one main cluster and suggested they may derived from a common ancestor (Figure 3). However, a whole genome sequence is needed to determine genetic pattern of Malaysian FCoVs. Phylogenetic tree constructed by neighbor-joining method showed the phylogenetic relations of the sequences in an unrooted-tree algorithm (Figure 4).

In conclusion, the present study indicated that males and young cats are more likely to be diagnosed with FIP. The homology of partial sequences of 3'UTR of FCoV isolates in Malaysia was shown to be higher than those from the other regions.

## Competing interests

The authors declare that they have no competing interests.

## Authors' contributions

SSA designed and coordinated the study and helped in draft correction. SSH carried out the molecular studies, performed the RT-PCR assay and sequence analysis and drafted the manuscript. MHB, ARO and NAZ participated in the sequence analysis and proof reading. LSF, NAR, HA and SHSH participated in the collecting of clinical samples. MAHI helped in lab works. All authors read and approved the final manuscript.
